# Effects of Oral Ginger Supplementation on the INR

**DOI:** 10.1155/2019/8784029

**Published:** 2019-06-11

**Authors:** Daniel Rubin, Vishal Patel, Eric Dietrich

**Affiliations:** University of Florida, Gainesville, FL, USA

## Abstract

Although there are competing options for anticoagulation, many Americans remain on warfarin. Concurrently, the use of alternative medicines is on the rise. With that, the potential for interactions between them is increasing. Ginger has gained popularity in its use. The literature has sparse recent information about the potential conflict between warfarin and ginger. This brief case report discusses the potential scope of the problem as well as the proposed pharmacokinetic and pharmacodynamic interactions between these two commonly used agents.

## 1. Introduction

It has been reported that up to 71% of American adults take some form of dietary supplement ranging from oral multivitamins pills to green tea [1]. Supplements are technically regulated by the Food and Drug Administration (FDA) under the Dietary Supplements Health Education Act (DSHEA). Supplements, however, are not proactively regulated. In addition, clinical trials that have proven health efficacy are often lacking, leaving physicians and healthcare providers unsure how dietary supplements may interact and change the pharmacokinetics and/or pharmacodynamics of a patient's medication regimen. There are numerous online databases that allow healthcare providers to query potential adverse drug reactions. While these databases have made counseling patients about dietary supplements easier, it is challenging to isolate and discuss every potential interaction with patients given the lack of clarity as to exact contents of many over-the-counter supplements.

Ginger (*Zingiber officinale*), which can now be purchased in the form of a garnish, beverage, oral supplement, soap, and oil, has become increasingly popular for its antiemetic properties and more recently for the symptomatic control found in patients with osteoarthritis and rheumatoid arthritis [2]. While there has been no direct advisory against the incorporation of ginger in the diets of patients who are anticoagulated with Coumadin (warfarin), there is a warning from the Food and Drug Administration (FDA) advising healthcare providers to be cautious in patients who are on warfarin and who also use ginger, garlic, and other dietary and herbal supplements [3]. This report highlights a case where an oral ginger supplement altered the anticoagulant effects of warfarin.

## 2. Case Report

Our patient is a 70-year-old Caucasian female with a past medical history significant for obstructive sleep apnea, osteopenia, restless leg syndrome, deep venous thrombosis, and cerebral vascular accident who is on long-term warfarin therapy. Her medication list included clonazepam 1 mg, metoprolol succinate 25 mg, paroxetine 10 mg, phenytoin 30 mg, rosuvastatin 20 mg, warfarin 7.5 mg, and warfarin 10 mg, none of which had been altered during the preceding month. She presented to our clinic for an international normalized ratio (INR) check after having a therapeutic INR of 2.7 one month prior. Her warfarin was dosed at 7.5 mg daily except 10 mg on Wednesdays.

During our visit with her, she was found to have an INR of 8.0, but she did not endorse bright red blood per rectum or melena, bleeding of her gums, hematuria, or epistaxis, and chest pain or shortness of breath. Since her last visit, one month prior, she began taking “Ginger Rescue,” a daily oral, chewable, 48 mg ginger supplement that had no other herbal or active ingredients. She did not report any other dietary changes in the previous month. Additionally, she did not endorse introducing any other supplements, outside of ginger, into her diet. Since a drug-drug interaction with rosuvastatin and warfarin is possible, we confirmed consistent compliance, as well as no dosing changes for both medications. We counseled our patient on holding 3 doses of her warfarin and stopping the ginger supplement. We advised our patient to return to our clinic 1 week later for an INR recheck. At this time, her INR returned to 2.6. She was subsequently advised to begin taking warfarin 7.5 mg daily.

## 3. Discussion

There are insufficient data present in the literature that clearly identifies how ginger interacts and alters the pharmacokinetics and pharmacodynamics of warfarin. In a case report presented by Lesho et al. in 2007, an interaction amongst ginger, warfarin, and intestinal and hepatic p450 systems was loosely postulated to be the cause of a supratherapeutic INR of 7 in a 76-year-old woman [4]. A second case report by Krüth et al. in 2003 presented a 76-year-old patient who began using ginger products and had a subsequent increase in her INR to 10 [5]. A prospective longitudinal study conducted by Shalansky et al. in 2007 concluded that the consumption of ginger was associated with an increase in bleeding risk in patients who were on anticoagulation; however, the study failed to show a significant interaction between supratherapeutic INR and ginger consumption [6]. Unfortunately, none of these studies specify the quantity of ginger consumed nor the mode of ingestion. Further, because dietary supplements are not regulated by the FDA, it is challenging to comment on the particular and exact contents of the oral supplement as an additional unidentified compound in the supplement could have contributed to, or completely caused, the elevated INR.

Contrary to what was reported in the above case reports, an open label, three‐way crossover randomized study conducted on healthy subjects by Jiang et al. in 2005 found that Blackmores Travel Calm Ginger (equivalent to 0.4 g of ginger rhizome powder) did not significantly impact blood's ability to clot [7]. While there equipoise regarding whether or not ginger impacts anticoagulation in warfarin patients, case reports like the one presented here and presented by Lesho and Krüth should remind us to be cautious when thinking about dietary supplements and the role they may have on the pharmacokinetics and pharmacodynamics of a patient's medication regiment. However, it should also serve as a reminder as to how quickly the anticoagulation effects of warfarin may be impacted. Our patient had consumed 48 mg of oral ginger supplement. After stopping the oral ginger supplement entirely and holding her warfarin therapy for 3 days, our patient's INR returned to a therapeutic level. Upon resuming warfarin at 7.5 mg daily, she remained in the therapeutic INR range. Despite the inconsistencies present in the literature regarding an interaction between ginger and warfarin, our patient experienced a supratherapeutic INR, which was mitigated by stopping the ginger, and demonstrated the importance of being hypervigilant in the age of dietary and herbal supplements for potential interactions with patient's medication regimens.
